# Plasma-Enabled Smart Nanoexosome Platform as Emerging Immunopathogenesis for Clinical Viral Infection

**DOI:** 10.3390/pharmaceutics14051054

**Published:** 2022-05-13

**Authors:** Seyyed Mojtaba Mousavi, Seyyed Alireza Hashemi, Ahmad Gholami, Masoomeh Yari Kalashgrani, Neralla Vijayakameswara Rao, Navid Omidifar, Wesley Wei-Wen Hsiao, Chin Wei Lai, Wei-Hung Chiang

**Affiliations:** 1 Department of Chemical Engineering, National Taiwan University of Science and Technology, Taipei City 106335, Taiwan; mousavi.nano@gmail.com (S.M.M.); vijayrao@mail.ntust.edu.tw (N.V.R.); weshsiao@mail.ntust.edu.tw (W.W.-W.H.); 2 Nanomaterials and Polymer Nanocomposites Laboratory, School of Engineering, University of British Columbia, Kelowna, BC V1V 1V7, Canada; s.a.hashemi0@gmail.com; 3 Biotechnology Research Center, Shiraz University of Medical Science, Shiraz 71468-64685, Iran; gholami@sums.ac.ir (A.G.); masoomeh.yari.72@gmail.com (M.Y.K.); 4 Department of Pathology, School of Medicine, Shiraz University of Medical Sciences, Shiraz 71468-64685, Iran; omidifarn@sums.ac.ir; 5 Nanotechnology and Catalysis Research Centre (NANOCAT), Institute for Advanced Studies (IAS), Universiti Malaya (UM), Level 3, Block A, Kuala Lumpur 50603, Malaysia

**Keywords:** viral infections, convalescent plasma, smart nanoexosomes, drug delivery

## Abstract

Smart nanoexosomes are nanosized structures enclosed in lipid bilayers that are structurally similar to the viruses released by a variety of cells, including the cells lining the respiratory system. Of particular importance, the interaction between smart nanoexosomes and viruses can be used to develop antiviral drugs and vaccines. It is possible that nanoexosomes will be utilized and antibodies will be acquired more successfully for the transmission of an immune response if reconvalescent plasma (CP) is used instead of reconvalescent plasma exosomes (CPExo) in this concept. Convalescent plasma contains billions of smart nanoexosomes capable of transporting a variety of molecules, including proteins, lipids, RNA and DNA among other viral infections. Smart nanoexosomes are released from virus-infected cells and play an important role in mediating communication between infected and uninfected cells. Infections use the formation, production and release of smart nanoexosomes to enhance the infection, transmission and intercellular diffusion of viruses. Cell-free smart nanoexosomes produced by mesenchymal stem cells (MSCs) could also be used as cell-free therapies in certain cases. Smart nanoexosomes produced by mesenchymal stem cells can also promote mitochondrial function and heal lung injury. They can reduce cytokine storms and restore the suppression of host antiviral defenses weakened by viral infections. This study examines the benefits of smart nanoexosomes and their roles in viral transmission, infection, treatment, drug delivery and clinical applications. We also explore some potential future applications for smart nanoexosomes in the treatment of viral infections.

## 1. Introduction

Recently, scientists have studied cells and found that, in addition to secreting soluble factors, cells produce a series of nano-vesicles called exosomes with a diameter of 40–120 nanometers. After the outer membrane (inner vesicle) fuses with the plasma membrane, they are secreted into the extracellular environment by the multi-vesicular endosome. Therefore, nanoexosomes are another method of cell communication [1,2,3,4,5,6]. Nanoexosome production was initially thought to be a method of disposing of intracellular wastes [7,8].

However, after that, the important role of nanoexosomes in natural and abnormal biological processes was proven [9,10,11]. These vesicles are part of the extracellular vesicle family. Extracellular vesicles are classified according to size and origin. Nanoexosomes are the smallest member of this family and are made through the endocytosis pathway. Numerous molecules are involved in their construction, intracellular transport and secretion. Numerous mechanisms have been identified in their construction and loading [12,13].

The membrane vesicles that most cells make and send out as extracellular vehicles (EVs) include a wide range of other membrane vesicles. EVs are divided into two types: [3] ectosomes and [14] exosomes, which differ in size, biogenesis and biophysical properties.

Smart nanoexosomes are formed when multivesicular bodies (MVBs) are secreted with the fused plasma membrane by the exocytosis of intraluminal vesicles (ILVs) [15,16,17]. With the further potential to enable complicated biological reactions, smart nanoexosomes are suitable diagnostic and therapeutic tools for a variety of diseases. Smart nanoexosomes, due to their biogenetic origin, can penetrate other cells without being targeted by the immune system or causing toxicity and can cause the penetration and delivery of functional cargo molecules [18,19,20,21,22].

Smart nanoexosomes are nanomaterials that have remarkable pharmacokinetic, bioavailable and biodistributive properties among others. Their characteristics include low toxicity and immunogenicity, which are very low in comparison to other parts of the body. It is thought that more smart nanoexosomes are released by cancer cells than by normal cells, which may help in the formation of tumors [23,24,25,26,27,28]. In recent studies [29,30], smart nanoexosomes appear to play many different roles in the development of viral diseases.

As an example, when smart nanoexosomes move through the body, they may help fight infections by activating natural or learned immunity and killing pathogens through apoptosis or other signaling pathways [31,32,33,34]. Even though it is not confirmed whether various types of smart nanoexosomes are made by various parts of the process of making MVBs, this is thought to be the case. What is unclear is the mechanism by which MVB is allowed to combine with the plasma membrane instead of dissolving in a lysosome.

Rab35 is one of the activities of special membrane trafficking machinery on which the release of smart nanoexosomes depends [35,36,37]. Many features of the generation of intermediate latent virions (ILVs) within the MVB and the envelopment of enveloped virions are shared by the two processes. Both processes require the induction of a membrane primordium, the introduction of a specific cargo and the cleavage of the membrane to allow for release of the cargo.

There has been a convergence in the use of the ILV generation machinery by viruses that are essentially unrelated to promote their own budding [20,38,39,40]. The aim of this review study was to investigate the plasma-enabled smart nanoexosome platform in emerging immunopathogenesis for clinical viral infections and to highlight future areas integrated in the work in this field.

In addition, this review covers the current research on smart nanoexosomes as viral carriers in clinical applications, treatments and drug delivery with a particular focus on viral infections to enable the use of smart nanoexosomes in the development of viral therapeutics in the future. We also evaluate the background of smart nanoexosomes, plasma-derived smart nanoexosomes, the clinical translation pathway, smart nanoexosomes as biosensors and challenges with smart nanoexosome therapeutics.

## 2. Background of Smart Nanoexosomes

In certain studies, differential filtration has been used to distinguish between large microvesicles that are caught by 0.65 micron filters and microscopic “exosomes” that pass by zero- and one-micron filters [41,42,43]. EVs with unique surface markers, such as substance A33 (GPA33) and EPCAM [44], have been found by researchers who used high-speed separation to find them.

These markers include lipid moieties that bind the toxin cholera, annexin V, or Shiga toxin [45,46] as well as the tetraspanins CD63, CD19 and/or CD81 [47,48]. Thus, many possibilities, including the reprogramming of nanoexosomes, indicate the “smart” nature of these vesicles as a means of treatment. The ability of nanoexosomes to home in on target cells/tissues without scaffolding, to combine therapeutic molecules within the lipid layer and to easily change the desired surface ligands can contribute to the “smart” behavior of nanoexosomes.

In addition, such approaches are ideal for programming nanoexosomes to respond to changes in the cellular/tissue microenvironment. Nanoscale single-cell genomic manipulation is an option for identifying and screening smart exosome clusters based on their regenerative intermediates, which can be a model for smart exosome engineering. Intrinsic features, stem cell modification, direct modification and the ability to load are some of the features that make nanoexosomes “smart” [49,50,51,52,53].

**Figure 1:**
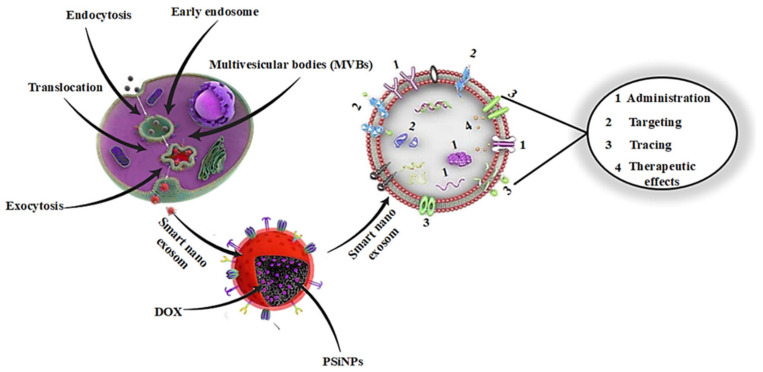
Background of smart nanoexosomes.

Current research distinguishes between different types of smart nano- exosomes depending on their origin. This categorization would ignore the properties of the different types of smart nanoexosomes and their practical uses. Smart nanoexosomes consist of a variety of molecules, as shown by high-throughput exosome studies, smart nanoexosomes contain a variety of molecules, including proteins, lipids, metabolites, mRNA [54], mitochondrial DNA [55], miRNAs [56] and alternative non-coding RNA masses [57]. Smart nanoexosomes also contain mitochondrial DNA [58], which is consistent with high-throughput exosome studies as seen in Figure 1.

Smart nanoexosomes have been discovered to be present in blood plasma/serum [58], secretions [59], breast cancer [60], cerebrospinal fluid [57], urine and liquid body matter [61]. Smart nanoexosomes are divided into two types: natural smart nanoexosomes and designed exosomes, regardless of whether they have been synthetically modified. Considering that the glandular system in animals has features inferior to those in conventional and tumor diseases, the exocrine system is divided into two groups: normal exocrine structures and cancer structures.

Macrophages, natural killer cells (NK), mesenchymal stem cells (MSCs), dendritic cells (DCs), human venous endothelial cells, T cells and B cells are some of the common cells used in the production of exocrine bodies [30,45,62,63,64], exocrine bodies can be formed by almost all common cell types. As tumor cells can release numerous exocrine bodies, certain antigens on their surface could mimic the characteristics of donor cells. Therefore, cancer research has shown great interest in tumor exosomes. Smart nanoexosomes for tumor growth—in addition to monitoring disease progression—play an important role in modulating immunity, tumor development and metastasis [65,66].

### 2.1. The Biogenesis of Nanoexosomes

Biogenesis nanoexosomes are endogenous bubble vesicles that are formed by germination in endosome division during endosome maturation, from primary to secondary endosomes in the form of multi-vesicular bodies [67]. Inside the cell, nanoexosomes are initially created by a process of infiltration into endosomal membranes to form molecular vesicles [68]. The formation of nanoexosomes begins with germination inside the endosomal membrane to form nanoexosomes vesicles in the cytoplasm. This process depends on the complex endosomal assemblage required for the carrier vesicles or ceramide sphingolipids [69].

Proteins ESCRT Hrs, CHMP4, TSG101, STAM1, VPS4 and other proteins, such as complex Syndecan-sytenin-ALIX, nSMase2, PLD2 and CD9, play important roles in controlling the mechanisms of biogenesis of nanoexosomes [23,54,70,71]. The dependent integration RAB GTPase of the nanoexosome of the parent cell releases the nanoexosomes produced into the extracellular space, where they can communicate with the receptor cells. Delivery of exosomal vesicles to the receptor cell can occur by interaction of the receptor ligand, pinocytosis/phagocytosis fusion with the cell membrane [67,72,73].

The formation of nanoexosomes vesicles during their formation shows some similarities with the exosome vesicles formed during lysosome formation, including the surface proteins of lysosomes, such as LAMP and CD63, which are also present in exosomal membranes [74]. Numerous external factors, including exocytosis, cell type, the presence or absence of cytokines, serum conditions and growth factors, affect the biogenesis of nanoexosomes. In addition, protein sorting, trans-acting mediators, nanoexosome sites and physical and chemical aspects regulate biogenesis [75].

### 2.2. Roles of Smart Nanoexosomes of RNA Viruses

The relationship between smart nanoexosomes and viruses is unclear. The pathway of exosome biogenesis overlaps significantly with the assembly and exit of numerous viruses. Many viruses use the exosomal pathway and interact with ESCRT proteins to enhance replication processes. Smart nanoexosomes contribute to infection and the evasion of the immune system as well as the evasion of the host immune system [6,9]. Human immunodeficiency viruses (HIV) are lentiviruses that belong to the Retroviridae family and cause acquired immunodeficiency.

Nef, for example, is a non-enzymatic auxiliary protein with many activities that HIV uses to infect host cells. Infected macrophages have been shown to produce more exosomes, and the expression of Nef appears to increase the amount of late endosomes/MVBs (responsible for exosome biogenesis) in a number of cell lines, including human T lymphocytes, according to CIIT. Smart nanoexosomes generated from viral-infected cells were found to contain virus genomic RNA, mRNA and miRNA. In the field of exosome production among RNA viruses, HIV-1 was the first RNA viral to be studied.

Integrated provirus transcription is performed by smart nanoexosomes produced from HIV-infected patients and/or HIV-1-infected cells that contain the virus transactivating response element (TAR) [76,77]. By reducing apoptosis, HIV proliferation in receptor cells is enhanced by TAR. Unspliced strains of HIV-1 RNA are adsorbed to smart nanoexosomes, and the presence of sequences within the 5′ end of the Gag p17 open reading frame is adequate for this recruitment.

**Figure 2:**
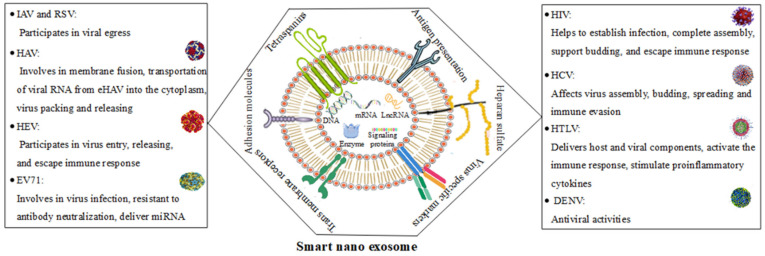
Smart nanoexosomes and their role in the pathogenesis of RNA viruses.

Single-stranded or double-stranded DNA does not cause exosome formation. Figure 2 shows smart nanoexosomes and their role in the pathogenesis of RNA viruses. Nucleic acids (such as non-coding RNA, DNA and mRNA), extracellular matrix proteins, metabolites and membrane proteins compose cargo molecules including smart nanoexosomes. The composition of smart nanoexosomes can vary greatly due to factors, such as their size, cellular origin, intrinsic cell biology and cellular microenvironment. Recently, the protection of RNAs against nuclease degradation as well as their release without side effects into the cytoplasm of target cells requires an effective delivery system. The development of RNAi-based therapies has been hampered by their rapid hydrolysis, bioavailability and bioavailability issues [78,79,80].

Currently, active targeting appears to be the most effective method to address this problem. Other nanovehicles have a superior targeting ability compared with other vehicles. First, the smart nanoexosomes show a weak household effect, which means that nanoexosomes derived from viral infections are preferentially taken up by viral infections. Secondary, modification of smart nanoexosomes derived from cell membranes is performed by different proteins or peptides and then actively targets virals. In RNA-based viral infections, smart nanoexosomes were found to be modified by a variety of target ligands, including tLyp-1, folic acid, iRGD peptide and T7-peptide [13,81,82,83].

### 2.3. DNA Viruses

Virus genetic material (such as DNA) or proteins encoded in the genome are among the items used to classify viruses. DNA viruses are small genetic units of intracellular parasites. In fact, they can only multiply by entering the host cell and using its resources and location for reproduction. DNA viruses use a variety of methods to replicate their genomes, such as RNA polymerases and DNA polymerases [84,85]. Table 1 shows the RNA and DNA species in smart nanoexosomes derived from virus-infected cells. Many DNA virals in a timed approach have the ability to control gene expression through a virus replication cycle.

The viral genome expresses “early” and “delayed” genes. The activation of the cell DNA machine is performed through the interaction of early genes with the cell. The accumulation of virions is possible through delay genes, which are mainly brown proteins. Amplification of all DNA viruses, except for poxviruses, occurs in the nucleus of infected cells. These virals generally depend on the DNA machinery in the cell. Due to the unavailability of DNA synthesis to replicate virus DNA, most host cells are inactive. The extracellular form of the viral is referred to as the “brain”.

The brain is a piece of nucleic acid that is enclosed in a protein coating called a capsid. Symmetry and flatness characterize the structure of the capsid. This brain can take over cells and bind to them. Consisting of a cell membrane, the capsids are covered in DNA viruses [86,87]. When the fibrous protein knob region binds to the host receptor, entry into the host cell begins. Subsequently, the formation of a network with integrated AV is possible by a pattern created in certain proteins.

Virion entry into the host into an endosome occurs due to the stimulation of adenovirus internalization through clathrin-coated cavities. The endosome becomes acidic after internalization, the topology of the virals changes, and the capsid components separate. This causes the virus to be released into the cytoplasm. Then, the virus travels to where the viral gene expression occurs (the nuclear pore complex) [88,89]. The immune system fights the innate immune responses that are incompatible with viral infections.

Intrinsic immunity is the starting line of defense against microbes and includes cellular and biochemical defense mechanisms that exist even before the infection enters the site, can respond quickly to the same infection and do not have the power to distinguish the exact difference between microbes.

Pattern recognition receptors (PRRs) are proteins that cells in the body’s innate immune system use to identify molecular patterns involved in pathogenesis. Activation of a sequence of signaling events by viral infection induces transcription of interferon type I (IFN) and proinflammatory cytokines. Recently, important information on the mechanisms of viral RNA identification and signaling pathways generated by RNA viruses has been provided through studies. Conversely, there is still no reliable information on the mechanism of triggering host antiviral defenses and the detection of DNA viral infection by host cells.

A total of 10 virus DNA sensors have been presented. Nevertheless, their use as common sensors for detecting DNA viruses in various types of animals and cells has not been confirmed [90,91]. Responses induced by IFN have no role in the immune responses to DNA. Cytosolic DNA triggers activation proinflammatory cytokines, such as interleukin (IL)-18 and IL-1ß, which are dependent on caspase-1. This pathway is facilitated by a protein called AIM2, which contains pyrin and a HIN200 domain (PYHIN).

Recent data from knock-out studies demonstrated the importance of AIM2 in host defense against DNA virals. One of the newest known PYHIN proteins is called IFI16. Immune responses to certain DNA viruses and viral DNA stabilization are among the cases in which IFI16 plays an important role. IFI16, as with AIM2, acts to achieve virus DNA by HIN domains. Activation of IFI16 causes the activation of inflammatory cytokines and ß-IFN production in response to cytosolically administered viral DNA or HSV-1 infection [23].

**Table 1:** RNA and DNA species present in smart nanoexosomes derived from virus-infected cells.

Virus	Genome	Features and Response	Ref.
HIV-1	ssRNA positive sense	Broad host range (non-dividing cells) Long-term, inducible expression	[92,93,94,95]
HIV-2		Chromosomal integration	
NDV	ssRNA negative sense	Replication in tumor cells Improved oncolytic vectors	[96,97,98]
HPV	dsDNA, papillomavirus	Double-stranded	[99,100,101]
B19V	ssDNA, parvovirus	dispensable for cell cycle arrest at phase G2/M	[102,103,104]
Herpesvirus	dsDNA	Risk of recombination with latently herpes simplex virus- infected cells	[105]

## 3. Plasma Derived Smart Nanoexosomes

The colorless part of the blood that lacks red blood cells and is more active in producing antibodies than other blood cells is called plasma. Cryosupernatant antibody-rich plasma and solvent/detergent-treated plasma are called virus convalescent plasma, and these are collected by producing neutralizing antibodies to the viral infection and donors recovering from the viral infection [106,107,108]. The transmission of intracellular blood from people whose viral load has decreased or who have recovered from a viral infection to people who are at risk for infection includes the inactive transfer of immune protection using convalescent plasma.

The reduction in deaths from severe influenza and related viruses is due to convalescent plasma therapy [109,110,111,112]. Therefore, among the factors that have a potential application against the side agents of convalescent plasma treatment, we can mention activated exosomes from immune-stimulated regulatory, suppressing T cells and M2-type macrophages [113,114,115]. Thus, it has been shown that M2-type macrophages and active smart nanoexosomes derived from immunosuppressed suppressors and regulatory T cells may have extraordinary side effects, including immunosuppressive effects [116,117,118].

Convalescent plasma therapy with an antigen-specific antibody on the exosome surface of plasma-derived immune cells has positive side effects. The use of viral convalescent plasma and epigenetically active lncRNAs also affects the receptor response to the viral. These plasma exosomes activated by the immune system may be responsible for, or inhibit, the beneficial effects of immune antibodies beyond those present in plasma. The inhibition of mRNA gene expression and mRNA conversion to a virus are among the applications of smart nanoexosomes [6,119,120,121].

## 4. Clinical Translation Pathway

### 4.1. Coronaviruses

**Figure 3:**
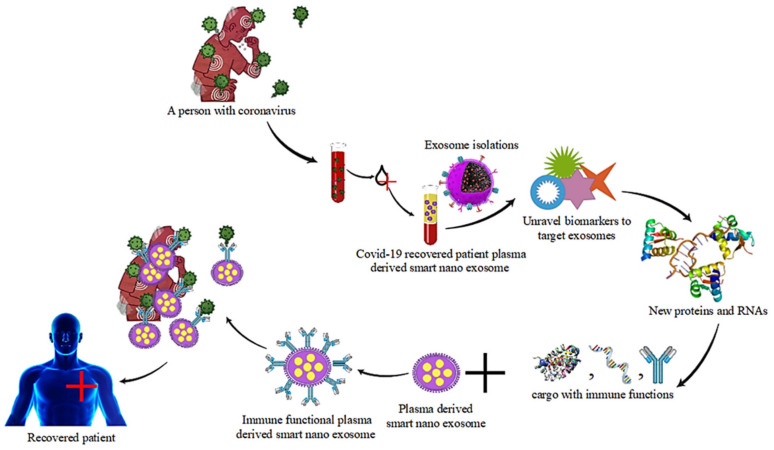
Recovery of a patient with coronavirus by plasma-derived smart nanoexosomes.

A number of molecular approaches are currently being developed or are already commercially available for the management and detection of viral diseases. The detection methods available today have both advantages and disadvantages. A gold standard for the detection of SARS-CoV2 is considered when RNA virus detection in a sample is performed by real-time polymerase chain reaction (RT-PCR). As can be seen in Figure 3, there are a number of approaches and methods, all of which have their advantages and disadvantages [122,123].

SARSCoV2 infection is classified into three stages: Stage one is associated with a low degree of symptomlessness or as long as no virus is detectable, stage two is associated with a nonsevere symptomatic phase in the presence of virus, and stage three is associated with a severe symptomatic phase in the presence of a high pathogen load. However, even though there are several diagnostic methods for detecting the virus, each approach has its own limitations that must be considered before use.

With recent advances, PCR-based technologies are unable to distinguish between the non-infective nucleic acid of the viral and the infected viral. Therefore, methods and substrates for detecting viral infections were immediately increased and developed. Smart nanoexosomes are released from virus-infected cells, and these smart nanoexosomes contain virus-derived miRNAs and proteins as well as viral receptors that allow the virus to enter recipient cells. According to reports, the detection of virus particles in dual membrane vesicles can be performed by culturing SARS-CoV in AT2 cells [77,124,125].

Important information about the differential secretion of cargo in cells infected with SARS-CoV compared to existing non-infected cells is obtained by studying exosomal cargoes. According to the research, smart nanoexosomes isolated from COVID-19- infected cells could contain specific proteins that can be detected and used as biomarkers for the disease as shown in Figure 3. These immune-activated smart nanoexosomes may have beneficial effects on plasma in addition to the immunologic antibodies they contain, or they may have inhibitory effects on plasma. Smart nanoexosomes are used to translocate microRNA (miRNA) in viruses to decrease the production of mRNA genes [126,127].

### 4.2. Influenza Viruses

**Figure 4:**
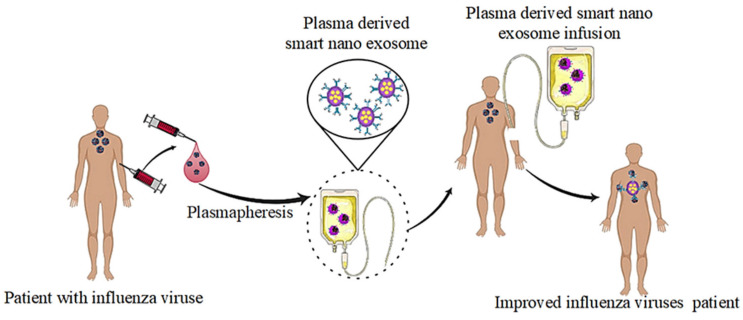
Improved influenza virus of a patient by plasma-derived smart nanoexosomes.

It was discovered that smart nanoexosomes produced by viral-infected cells, including a mixture of influenza virus and host cell components that have the ability to influence the responses of receiving host cells as shown in Figure 4. The researchers concluded that smart nanoexosomes were able to stimulate inflammatory responses in the lungs when they were released into the respiratory tract during the acute phase of influenza infection, which in turn triggers the production of innate immune cells and the production of proinflammatory cytokines.

Sialic acids associated with α2,3 and α2,6 are key receptors that are expressed through airway-releasing smart nanoexosomes and are also used by the influenza viral to become part of the target cells. Scientists have allowed either salivary glycoproteins or glycolipids superficially expressed by smart nanoexosomes to recognize the influenza virus’s individual hemagglutinin glycoprotein. They also equate sialylated cell surface receptors that are essential for host cell infection and HA binding. Studies have shown that influenza viral infection can be neutralized by airway exosomes.

According to other studies, reducing the severity of influenza infection in animals is possible by intravenously administering sialylated nanoparticles to mice [128,129,130]. The researchers believe that the internal smart nanoexosomes produced in the respiratory tract likely function through a mechanism similar to that used in viral infections, such as the influenza virus. One of the best treatments for diseases caused by severe insect contamination is the remarkable result of synthetic smart nanoexosomes, such as nanovesicles containing sialic acids.

It is also possible that the same approach will work for other virals, such as COVID-19 and rotaviruses, which continue to use sialic acid as a recipient to infect host cells. Scientists report distinct biological activity by which exosomes, during the release of influenza virus infection into the respiratory tract, are able to prevent the spread of the virus and have the potential to help the inherited antiviral immune response.

Smart nanoexosomes from the respiratory tract have antiviral properties. A new approach to promoting smart nanoexosomes or their synthetic derivatives by understanding how they modulate disease progression can be developed in influenza status and management, which remains a major threat to the global energy industry. The intranasal administration of sialylated nanoparticles to mice has been shown to reduce the severity of influenza infection [131,132,133].

### 4.3. HPV

A comparison of smart nanoexosomes generated by HPV and HPV HNSCC cell lines has shown that this is the case. These smart nanoexosomes have been shown to transport proteins that correspond to the molecular and functional characteristics of an individual’s tumor cells, thereby, providing the basis for this association. HPV or HPV cancer cells may be surrogates for smart nanoexosomes that mimic the cells of the individual, suggesting the effects of these smart nanoexosomes on tissues and uninjured cells.

A comparison of smart nanoexosomes derived from HPV cell lines and HPV with the reprogramming of HPV exosomes was performed using an experimental model with reprogramming of exosome-stimulated immune cells by HPV exosomes containing E6 and E7. Smart nanoexosome analysis, which results in unique sensitivity to antitumor therapy and better overall outcomes, reveals biochemical differences between HPV tumor cells and HPV [134,135,136,137,138]. These smart nanoexosomes inhibit the activity of CD4+ and CD8+ T lymphocytes.

Nevertheless, HPV exosomes had a deleterious effect on the formation of DC and the expression of APM components. In contrast, HPV exosomes not only did not inhibit the expression of APM fragments but also regulated the expression of co-stimulated CD80 and CD83 molecules in iDCs. Based on previous and current results, it can be concluded that the interaction of exosomes with receptor cells and/or their internalization has a significant role in the quality of the response to smart nanoexosomes [139,140,141].

As smart nanoexosomes from the plasma of HNC patients have been shown to contain immunosuppressive proteins, separate HPV and HPV exosomes have also been shown to be identical in morphology, variation and immunosuppression. Both HPV and HPV exosomes accurately downregulated the activities of activated T cells; therefore, it was not surprising that this was the case. However, we hypothesized that the contents of the smart nanoexosomes of virus-infected cells were more likely to be altered than the contents of smart nanoexosomes produced by uninfected cells.

**Figure 5:**
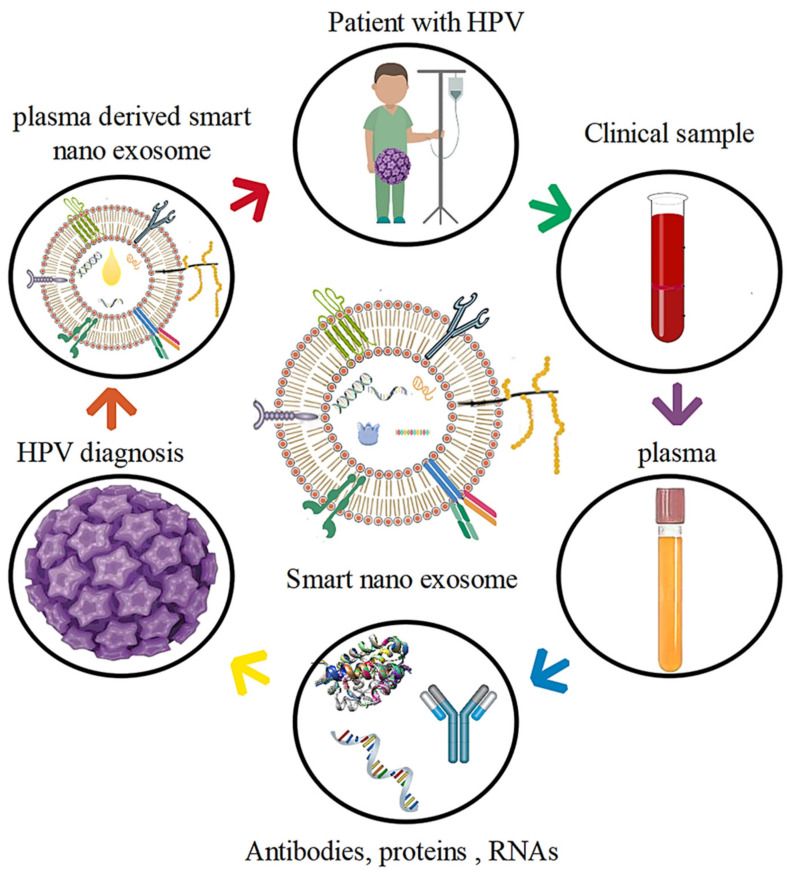
The treatment cycle of a patient with HPV by plasma-derived smart nanoexosomes.

HPV and HPV exosomes exhibited different protein properties because HPV HNC cells produce exosomes that lack E6/E7 proteins and other important molecules. As viral antigens produce strong immunity, it can be said that in experiments with human T lymphocytes, smart nanoexosomes produced by HPV cancer cells that contain E6 and E7 proteins are immune-stimulating. In these types of exosomes, excitatory molecules, such as OX40, OX40 L and HSP70 were found to be more than usual as shown in Figure 5 [142,143].

Instead, these smart nanoexosomes decrease the death of human T cells that have been activated. Research has shown a direct effect of the ratio of immunosuppressive proteins to stimuli in the exosome membrane on its ability to stop suppressing T cells.

The superiority of inhibition of T cell activation by surface receptors by cyst-derived smart nanoexosomes has been demonstrated by the presence of FasL, LAP-TGF and possibly other tumor-derived immunosuppressive proteins in HPV that counteract concomitant stimulation. In tests with activated human effector T cells, smart nanoexosomes identified as HPV or p16 reports were equally efficient at suppressing the immune system or inducing cell death in HPV or HPV exosomes derived from patient plasma. Immunosuppressive agents include deficiency of concomitant stimulatory proteins and cyst-derived exosomes with high inhibitory ligands [144,145].

### 4.4. Hepatitis and HIV

Several determinants and host factors are associated with the long-term persistence of hepatitis B virus disease. Chronic hepatitis B, liver cirrhosis and abnormal growth of liver cells are caused by the hepatitis B virus (HBV). Chronic HBV infection poses a health risk and incurs significant social costs, although HBV therapy can effectively prevent the spread of the virus. Moreover, the processes behind the diminished immune response and long-term infection caused by HBV are not well understood.

The role of smart nanoexosomes in HBV infection has not been elucidated, although the ability of smart nanoexosomes to transmit HCV between cells has long been known [79,81,146]. According to the results of the study, RNA and proteins of HBV were held together by the antitoxin of smart nanoexosomes from CHB patients. It was suggested that core HBV contains cccDNA, which has a lower copy number than other HBV DNA or RNA.

On the other hand, retroviruses are probably the only viruses that can hijack the exosomal machinery, grow in MVBs and then hide in exosomes. It is possible that HBV cccDNA is found in the cytoplasm of hepatic parenchymal cells in people who have CHB. This could lead to the unfolding of HBV cccDNA in smart nanoexosomes [147,148]. In the innate immune response to fiery infections, NK cells are the key effector cells, and the failure of NK cells allows fiery infections to persist. Researchers have found evidence of decreased cell cytotoxicity and IFN levels in patients with CHB, suggesting that NK cells may be damaged.

Hosts infected with HBV have increased levels of immunosuppressive determinants, such as TGF-, which contributes to NK cell failure. HBV-derived smart nanoexosomes have been shown to evade cytolytic and IFN-producing NK cells, demonstrating that HBV can modulate NK cell function through exosomes. It has been reported that HBV nucleic acids can be detected in smaller NK cells using PCR preparations or deep-sequencing studies of the HBV genome, although their efficacy is still controversial [3,149,150]. The biogenesis of nanoexosomes and the cell types that release the nanoexosomes determine the exosomal content.

The study of nanoexosomes secreted by HIV-1-infected cells is complicated by the fact that nanoexosomes share many features with HIV-1, including biophysical and molecular properties, biogenesis and uptake mechanisms. The density of nanoexosomes ranges from 1.13 to 1.21 g.mL^−1^, whereas the density of HIV-1 ranges from 1.16 to 1.18 g.mL^−1^ [151]. HIV-1 is slightly larger than exosomes, with the diameter of the virus ranging from 100 to 120 nm, while the diameter of exosomes is 40–100 nm [152]. In addition, the production of HIV-1 is possible through the same pathway of smart nanoexosome biogenesis [36].

Interaction with a number of cellular factors, such as TSG101 and Alix, is called HIV-1 germination, which play an important role in exosome biogenesis [153]. The convergence of exosome biogenesis and HIV-1 indicates that HIV-1 products (such as proteins and RNA) causes exosome products to become infected from HIV-1-contaminated fluids or to become encased within the exosomes. Isolation of HIV-1 particles from exosomes is accomplished, through immunoaffinity approaches and iodixanol density gradients, which are among the most intense purification strategies [152,154].

**Figure 6:**
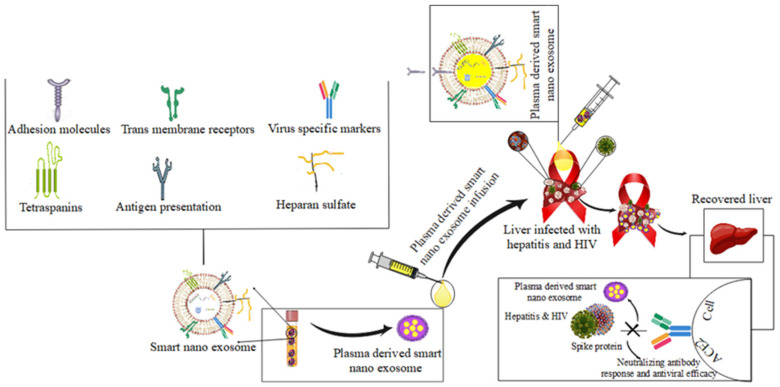
The mechanism of treatment of hepatitis and HIV by plasma-derived smart nanoexosomes.

Cell-to-cell proliferation and the exosome Trojan hypothesis of HIV-1 accumulation are due to striking similarities in HIV-1 in particular and the biogenesis of smart nanoexosomes and enveloping viruses in general [155]. Therefore, the evolution of uptake pathways for the formation of infectious virus and HIV-1 for the use of exosome biogenesis and the entry of env-independent virus is suggested. The mechanism of treatment of hepatitis and HIV by plasma-derived intelligent nanoexosomes is shown in Figure 6.

## 5. Smart Nanoexosomes as Biosensors

All molecules in smart nanoexosomes can potentially be used to diagnose disease. Smart nanoexosomes carry rich sources of potential biomarkers; the secretion of smart nanoexosomes into the extracellular space provides a good opportunity to examine body fluids, such as blood, urine and malignant ascites [12]. Smart nanoexosomes are widely present in patients with lung cancer, breast cancer, melanoma, etc. [156]. The dual role of smart nanoexosomes as biomarkers and messengers has provided opportunities for researchers to measure the spatio-temporal state of cells and take a closer look at the role of smart nanoexosomes in medicine [157].

Isolation of smart nanoexosomes and identification of their contents has led to the use of smart nanoexosomes as biomarkers for pathological conditions or the severity or stage of a disease. Smart nanoexosomes are widely used as biomarkers in cancer diseases. These studies are not limited to cancer, and similar studies have been performed on the proteome of exosomes of other cells and biological fluids. Study and understanding of the role of smart nanoexosomes in the cardiovascular system in cardiovascular physiology has led to the discovery of exosomal biomarkers in cardiovascular disease [158].

In addition, the miRNA expression pattern of smart nanoexosomes can be used as biomarkers for the early detection of various cancers; For example, in the case of breast cancer, which is one of the most common malignancies, researchers have suggested that the expression of exosomal miRNAs, such as miR-1246, miR-10, miR-21, miR-181 and miR-373 can be used as biomarkers in the early stages of cancer progression [159,160].

In addition to traditional methods, such as flow cytometry, nanoparticle tracking analysis (NTA), enzyme-linked immunosorbent assay (ELISA) and western blotting, different biosensing platforms have been developed for the analysis of smart nanoexosomes by targeting their surface proteins using the corresponding antibodies aptamers or antibodies. Thus, the surface plasma resonance (SPR) biosensor has attracted widespread attention as a fast, label-free and real-time diagnostic device.

Nevertheless, the SPR biosensor encounters unfavorable conditions for the detection of smart nanoexosomes: (1) the low mass and small size of smart nanoexosomes cannot cause obvious signal differences, which leads to the inevitable need for signal amplifiers; and (2) the collected smart nanoexosomes are always mixed with the serum-free target proteins, which results in the production of a false positive signal that reduces the accuracy of the results [161].

## 6. Challenges with Smart Nanoexosome Therapeutics

Smart nanoexosomes are attracting increasing interest as potential growing stars because of their multifaceted functions, ranging from remedies to drug transport, automobiles and detections. Notwithstanding these benefits, there are still quite a few clinical studies looking at the use of these nanovesicles. This can be due to the many challenges associated with nanoexosomes, which require additional investigation. One of the essential challenges is to keep their balance and practicality for a period of time.

Nanoexosomes, in contrast to MSCs, are much more powerful and can survive at −80 °C for extended periods of time. At some point in storage, freeze–thaw cycles can cause the exosomes to clump together. Moreover, keeping the temperature all the way through managing and transport is combined with an obstacle to their application in translation. Therefore, different maintenance strategies should be examined to improve their transport and equilibrium [162].

The suitability of freeze-dried smart nanoexosomes at room temperature has also been assessed by several studies to overcome these challenges. One of the reliable methods for proteins and nucleic acids, which includes molecules that are highly unstable, is freeze-dried formulations. Therefore, their application is increased by reducing prices by shortening cold-chain maintenance during transportation, thereby, increasing the service life and reducing storage needs [163,164].

However, during the freeze-drying process, the degradation of the exosome cargo and their accumulation has become a problem. This problem is solved by the addition of numerous stabilizers, such as sucrose, trehalose and glucose, which replace the hydration sphere around smart nanoexosomes during the freeze-drying process, preventing their aggregation and maintaining their membrane integrity [165,166]. Considering the above challenges and the usefulness of the freeze-dried methodology, we tend to compare the freeze-dried formulation of smart nanoexosomes with the non-freeze-dried formulation.

We prefer to select Wharton’s jelly-derived MSCs as the source of smart nanoexosomes because they have high immunomodulatory properties and can be used in viral infections, such as diseases [167]. Therefore, the advantage of this system in pertinence of nanoexosomes and enhancing the supply has been shown, which confirms the application of nanoexosomes in biomedical research. Smart nanoexosome therapeutics, such as sources, cargoes and loading mechanisms as well as the observed effects for smart nanoexosomes are shown in Table 2.

## 7. Conclusions and Perspective

This study provides an overview of the importance of smart nanoexosomes and subgroups of extracellular vesicles in viral infection as well as the methods by which they cause viral infection. Smart nanoexosomes are a type of nano-extracellular double vesicle that arises in the endosomal region of most eukaryotes and are found in the cytoplasm of many bacteria. Smart nanoexosomes have multiple biological purposes: they transfer their cargo to other cells, and they act as mediators of cell communication and regulators. The structure of nanoexosomes depends on the origins of the cells and tissues.

As a result, they may have various compositions in different pathological circumstances. Smart nanoexosomes are affected by many processes that are useful for many purposes, including being abducted by many viruses. Finally, the secretion of viral particles, the regulation of the production of virions and the activation of their capsid packaging by viruses are accomplished through hijacked exogenous biogenesis systems. Therefore, smart nanoexosomes are used as exogenous viral miRNAs for transmission to non-infected cells and/or nanocarriers of viral proteins.

Smart nanoexosomes have the potential to be important for a variety of biological functions, including vehicles to transport many components from one cell to another, modulating immunity and cellular communication. In addition, viruses also use smart nanoexosomes similar to other viruses to be transported for intra-host spreading and viral reproduction. Therefore, smart nanoexosomes can be suitable candidates for the preparation and development of many viral vaccines for use in the treatment and prevention of many pandemic infections, such as COVID-19, HPV, HIV, influenza and hepatitis.

Due to the different functions of smart nanoexosomes in biological and pathological processes, these small membrane vesicles have attracted widespread attention in the last decade. Smart nanoexosomes have created a new therapeutic approach for the transfer of biomolecules and drugs and can transfer various compounds, such as proteins, lipids, nucleic acids and drugs.

Some of the most interesting advantages of smart nanoexosomes that have received increasing attention are that they can be engineered, different compounds can be placed inside them, and their specificity can be increased by transferring specific exosomal receptors. We hope that, in the not-too-distant future, smart nanoexosomes will be useful to develop different vaccines to treat many diseases, particularly cancer.

## Figures and Tables

**Table 2:** Smart nanoexosome therapeutics, such as the sources, cargoes and loading mechanisms as well as the observed effects for smart nanoexosomes.

Smart Nanoexosome Source	Cargo and Loading Mechanism	Effect Observed	Ref.
Mesechymal Stem Cell	Anti-miR-9 (Transfection)	Reversal of chemoresistance	[168]
	miR-133 b (Transfection)	Suppression of progression	[169]
	Paclitaxel (Incubation)	Growth inhibition of human pancreatic adenocarcinoma cell	[170]
Dendritic Cell	BACE1 siRNA (Electroporation)	Knockdown of specific gene after specific siRNA delivery to the brain for AD	[171]
	Doxorubicin (Electroporation)	Specific drug delivery to the tumor site and inhibited tumor growth	[172]
HEK293T	BCR-ABL siRNA (Transfection)	Overcome pharmacological resistance in CML cells	[173]
Mouse lymphoma cell	Curcumin (Mixing)	Increase anti-inflammatory activity	[174]

## Data Availability

All data generated or analyzed during this study are included in this published article.
